# Toward accurate prediction of apple firmness and brix across countries, seasons and cultivars with hyperspectral imaging

**DOI:** 10.1007/s44187-026-01072-y

**Published:** 2026-06-10

**Authors:** Haidee Tang, Yuan Liu, Issam Boukhennoufa, Wangyang Yu, Xiangming Xu, Xiaojun Zhai

**Affiliations:** 1 Niab, New Road, East Malling, West Malling, Kent ME19 6BJ UK; 2 School of Computer Science and Electrical Engineering, University of Essex, Wivenhoe Lodge Boundary Road, Colchester, Essex CO4 3SQ UK; 3 School of Artificial Intelligence and Computer Science, Shaanxi Normal University, 199, South Chang’an Road, Yanta District, Xi’an, 710062 Shaanxi China

**Keywords:** Apple maturity, Vision transformer, Hyperspectral imaging, Multi-seasonal data, Multi-cultivar data

## Abstract

Traditional apple maturity assessment methods are destructive and time- and labour-intensive, yielding only population-level approximations. Hyperspectral imaging provides a non-destructive alternative to assess individual fruit, but progress has been constrained by the lack of large, diverse datasets that support robust model generalisation. This study presents a multi-cultivar, multi-season, multi-country hyperspectral apple dataset to enable generalisable prediction of soluble solids content (Brix) and firmness. Using this dataset, we adopt an iterative modelling framework to evaluate deep learning architectures, image resolutions, cultivar encoding, seasonal effects, and feature-specific models. Wavelength and spatial region importance were also analysed. The best predictive performance was achieved using Vision Transformer (ViT) models trained on edge-cropped 40 *× * 40 pixel images with explicit cultivar encoding, with Brix and firmness modelled independently. Although seasonal specificity was observed, models trained across all three seasons achieved the strongest overall performance. A 50% reduction in spectral wavebands did not compromise prediction accuracy. Key wavelength ranges contributing to Brix and firmness prediction were identified across the visible–near-infrared spectrum. Spatial regions were unimportant for Brix prediction but showed relevance for firmness. The optimised ViT model achieved firmness prediction performance comparable to previous studies (RMSE = 0.76 kgf, R$^{2}$ = 0.63), while Brix prediction accuracy was lower (RMSE = 0.91 $^{\circ }$Brix, R$^{2}$ = 0.75), likely reflecting increased biological and environmental variability captured in the dataset. Overall, this work demonstrates that hyperspectral imaging combined with deep learning and large, diverse datasets enables robust, non-destructive prediction of apple quality attributes across production conditions.

## Introduction

Quality apples have been linked to the timing of harvest, particularly harvesting at their optimum maturity. Traditional apple harvest prediction methods are essential to ensure high-quality apples. Traditional methods require the progressive measurement of biochemical constituents through the use of puncture tests for firmness, refractometry for sugar content, starch pattern index (SPI) readings for starch measurements and ethylene sampling via chromatography. Although these methods are the gold standard in the fruit industry, the tests are time-consuming, labour-intensive and rely on destructive sampling. Moreover, ethylene measurements require expensive specialised machines and trained staff to operate. Imaging has emerged as an efficient, non-destructive method to predict fruit maturity, but many fruit studies use a limited number of samples to train their models and are often difficult to interpret. In this study we aim to train a deep learning model with our broad dataset, encompassing multiple cultivars, growing seasons, and countries to accurately predict key maturity features (Brix, firmness and starch). Moreover, we aim to interpret the models to extract important wavelength and significance of apple region information.

Previous studies have shown that simpler imaging data can be analysed and interpreted to predict fruit physiological traits. Visible and near-infrared (VIS/NIR) spectroscopy has been a common area of study due to the obvious green-to-red (or yellow) colour change in the apple development process and the reflective soluble sugars at NIR. Water is also highly absorptive in NIR [13], and can introduce complexity and noise into the data—particularly relevant given that water makes up approximately 80% of apple content [27]. Due to this noise, assigning wavelengths to specific functional groups (e.g. –OH or –COOH) is ineffective. Most studies identify significant spectral indices—ratios between specific wavelengths—that correlate with the presence and concentration of biological compounds or physiological traits. Some studies observe chlorophyll degradation using the difference in absorption ratio between 670 and 720 nm (IAD) [12, 14, 23, 35], and others look at the ratio of anthocyanin (R800/R678) or carotenoids (R550/R700) and chlorophyll [11]. IAD also shows some correlation with firmness [14], Brix [14] and starch degradation [23]. However, using IAD to predict maturity parameters may not be consistent year-to-year [23].

The most common method of interpreting spectral data has been multivariate statistical methods, which help extract informative wavebands from spectral data. Methods such as Partial Least Squares Regression (PLSR), Principal Component Analysis (PCA), Multiple Linear Regression (MLR) and their variants [5, 10, 28] are used to address the high collinearity of spectral wavelengths and to model fruit maturity traits effectively. Wang et al. [28] found wavelength indices for chlorophyll, carbohydrate and water absorbance. The most significant wavelengths include the normalised difference spectral indices between 767 and 737 nm for Brix and 764 and 820 nm for firmness. On the other hand, Li et al. [10] identified 14 wavelengths in the infrared region (interesting spectra were found between 907 and 1107 nm) to define Brix. Although these models are highly interpretable, a key limitation is their inability to capture complex non-linear relationships between spectral features and maturity traits. Therefore, approaches capable of modelling such non-linear relationships should be considered.

Hyperspectral imaging has been successful in fruit detection and classification [24], maturity estimation [4] and disease detection [31]. Hyperspectral imaging has been used for maturity trait predictions in fruits such as apple [5, 28], mango [16], tomato [7], cherry [29] and grapes [18]; grains and seeds such as, rice [15], cocoa bean [17]; and root vegetables such as sweet potato [1]. It has successfully been analysed with deep learning models for learning complex patterns [3, 20]. The application of deep learning models to apple spectral data remains relatively understudied. Pourdarbani et al. [20] used an Artificial Neural Network (ANN) with Simulated Annealling algorithm to classify the ripeness of apples. This study was focused on 170 Fuji fruit, making the number of samples in each group limited. However, they achieved a high accuracy for the classifiers (93−99.6%) using spectral bands from the visible and NIR region. Another study [34], evaluates the predictive performance of a model trained on bagging types on 307 apples. The study used two machine learning models, ELM - a feed forward neural network and BPNN - a neural network model with error backpropagation. Their SSC predictions were 0.59 and 0.62 RMSEP using ELM and BPNN, respectively. They performed better than simple regression methods (0.2−0.86 RMSEP) predicting SSC. Another study used ANN, Decision Trees and K Nearest Neighbours to predict firmness and SSC trained on 100 Pink Lady fruit, achieving RMSEs of 0.91, 0.88 and 0.78 for firmness and 0.68, 0.81 and 0.80 for Brix, respectively [3]. When employed, deep learning models often lack interpretability, offering limited insight into the importance of key spectral indices due to their black-box nature. Moreover, despite the importance of large sample sizes when training deep learning models, only a limited number of studies utilise large-scale dataset that exceed 300 apples [3, 34].

In the present study, we are the first to collect and train a deep learning model on an extensive apple hyperspectral dataset comprising of 5756 apples, sourced from orchards in both the United Kingdom and New Zealand. This dataset also covers 6 cultivars across 3 growing seasons. This dataset provides a robust foundation for predicting key maturity traits in apples from many cultivars and different growing conditions. We also explore the use of Shapley values to explain the significance of each wavelength, as deep learning models are ’black-box’ models and lack interpretability. In this study, we evaluate (1) four distinct deep learning model architectures: 2D Convolutional Neural Network (2D-CNN), a 3D Convolutional Neural Network (3D-CNN), a hybrid CNN-transformer model, and a Vision Transformer (ViT) model; (2) the wavelengths important for brix, firmness and starch predictions; (3) regions of the apple which are significant; (4) the cultivar and seasonal effects on model predictions.

## Material and methods

### Fruit samples

Fruit of six apple cultivars were collected from Kent, United Kingdom and Hawke’s Bay and Nelson, New Zealand in three harvest seasons. Fruit were harvested during the harvest seasons from NZ (February to April) in 2023 and 2024, and from UK (September to October) in 2024. In England, Cox, Braeburn, Fuji, Gala and Golden Delicious were picked from East Malling (51$^{\circ }$17*\prime * 07.0$\prime \prime$ N 0$^{\circ }$27*\prime * 13.2$\prime \prime$ E) and additional Gala, Braeburn, Cox and Jazz were sourced from orchards around Harbledown, UK (51$^{\circ }$16*\prime * 33.5$\prime \prime$ N 1$^{\circ }$2*\prime * 28.4$\prime \prime$ E). All cultivars, except Jazz, were picked from orchards at Plant and Food (PFR) Research Hawke’s Bay (39$^{\circ }$39*\prime * 37.4$\prime \prime$ S 176$^{\circ }$52*\prime * 45.9$\prime \prime$ E). Additional Gala and Fuji were picked from a commercial orchard (39$^{\circ }$36*\prime * 11.5$\prime \prime$ S 176$^{\circ }$49*\prime * 20.9$\prime \prime$ E), Golden Delicious was picked from a local grower (39$^{\circ }$36*\prime * 54.2$\prime \prime$ S 176$^{\circ }$50*\prime * 52.1$\prime \prime$ E), and Braeburn and Fuji from PFR Nelson (41$^{\circ }$06*\prime * 49.7$\prime \prime$ S 172$^{\circ }$59*\prime * 04.3$\prime \prime$ E) in 2023 and 2024. All apples were picked in the morning, imaged, then the firmness, Brix and starch of each fruit were measured within 36 h of harvest at ambient temperature. A total of 5756 apples were harvested (Table 1).

**Table 1:** Image and apple count per season split by cultivar. Numbers represent pre-filtering counts

Cultivar	NZ2023		NZ2024		UK2024	
	Apples	Images	Apples	Images	Apples	Images
Braeburn	47	32	350	236	804	464
Cox	248	180	0	0	119	72
Fuji	811	472	524	360	280	160
Gala	463	356	424	292	850	492
Golden delicious	132	104	111	76	574	328
Jazz	0	0	0	0	19	12
Total	1701	1144	1409	964	2646	1528

The equipment reported for each maturity feature was used, as follows, in the UK and NZ, respectively. Firmness was measured using a fruit texture analyser (Llyod LRX, UK and GÜSS, South Africa) with an 11 mm diameter probe to a depth of 8 mm. Two regions (approximately perpendicular to each other) along the equatorial region of the fruit were punctured after peeling away the skin. The force at maximum depth was used in this study. Atago refractometers (portable benchtop palette series, model PR-32*α * and pocket PAL series, model PAL-1) were used to measure the apple juice collected from the puncture sites made during the firmness process. The refractometers were calibrated at the start of each sampling day using distilled water. Lastly, the starch percentage was measured by cutting the apples in half horizontally then a potassium iodine solution was applied to one half of the apple by dipping them in a 1% w/v iodine and 4% w/v potassium iodide solution or spraying with 1% w/v potassium iodide and 0.25% w/v iodine. The apples were left to stain for 30 min (Per comms) before recording the staining percentage. The firmness readings from both sides of each fruit were averaged to create an average firmness reading.

### Obtaining fruit images and initial image processing

**Fig. 1:**
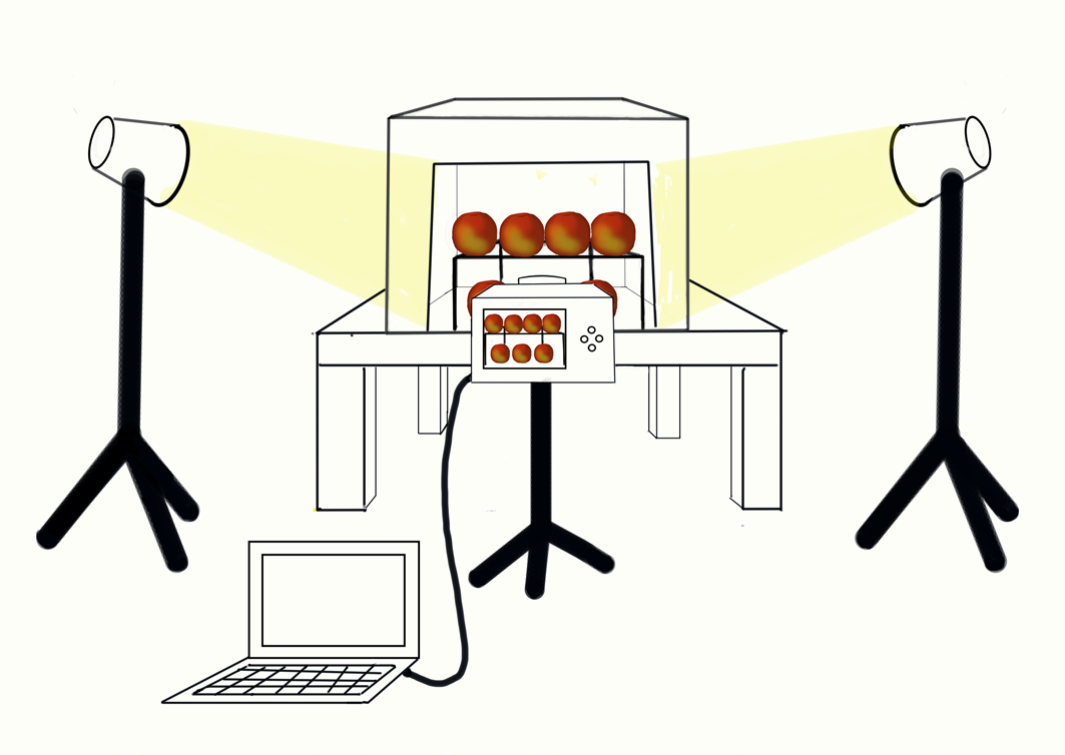
Schematic of the hyperspectral imaging equipment setup. Two lights on stands were set up on either side of a camera on a tripod, facing the light diffusion tent containing the apples. The lights and tent formed a triangle with sides of *∼ *1 m length

Images were taken using two different Specim IQ hyperspectral cameras of the same model (Specim Imaging Ltd., Oulu, Finland) using the same settings [2]; one was in New Zealand and the other was in the United Kingdom. The spectral range of the cameras was 400–1000 nm, with 204 spectral bands at 7 nm resolution. Two 750 W tungsten halogen lights (ARRILITE 750 Plus, ARRI, Germany) were each positioned approximately 1 m apart, forming a triangular setup with the photo shooting tent. This setup ensured little shadow was cast on the apples. The tent diffused the light to avoid overexposure caused by direct light on the waxy layer of apples. The camera was set in front of the tent (Fig. 1). The camera set-up and calibration were followed according to the manufacturer’s manual. The reflectance was generated by correcting with white and dark references to reduce background noise. Images were captured following appropriate integration times.

In a dark room, four images of each apple were taken by rotating the apples by 90$^{\circ}$ on the horizontal axis to attain four equatorial images. These were processed at room temperature before the maturity assessments. Depending on the size of the fruits, each image contained between 2 to 8 apples arranged in two rows. A total of 3636 images were taken (Table 1). The image metadata was inputted during the imaging process, ensuring the date, cultivar and fruit numbers were recorded. Some apples may not have images from all four sides due to human errors during the collection process.

### Image processing

Since multiple apples were presented in the images, object detection was done using Segment Anything Model 2 (SAM2) [22] on the RGB images generated by the hyperspectral camera. Occasionally, SAM2 would not detect all the apples within the image. To ensure that we retained the correct number of actual apples and those detected by the image detection AI, we counted the number of bounding boxes detected and matched it to the count of fruit noted in the metadata. When the numbers did not match for that image, the image and the maturity data for those apples were removed from the final dataset. 2.75% of the images were filtered out due to mismatches between the detected and actual number of apples in the image. Valid masks were applied onto the hyperspectral images so that any pixel coordinates for any wavelength outside of the segmented area is zeroed.

The apple images within the bounding box coordinates (apple and zeroed background) were divided into equal parts before locally averaging the pixels, forming either 30-by-30 or 50-by-50 pixel images. This method reduces the size of the tensors and retains spatial information. To further localise just the centre of the images, 5 pixels were removed from each edge of the generated images, thus forming 20-by-20 or 40-by-40 pixels images. Due to the curvature of apples and lower quality of pixel values around the edge of the fruit, removing the edges may improve model accuracy [28]. The spectral data, 204 contiguous bands, made up the third dimension of the image. In addition to the hyperspectral data, each sample was labelled with its corresponding cultivar information using one hot encoding so that it is trainable by machine learning algorithms. The six cultivars in the study were encoded into a binary format and integrated into the last layer of the images, increasing the third dimension from 204 to 210 channels, unless otherwise stated.

**Fig. 2:**
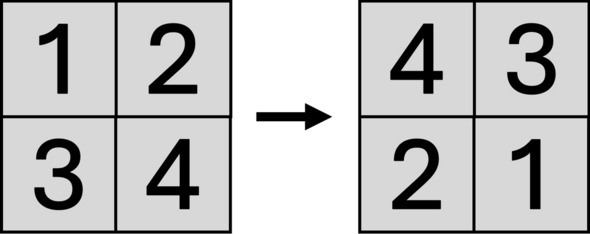
Example of a swapped quadrants image. The four corners were swapped diagonally to simulate whether apple regions are important

The data was first split into their respective cultivars before randomly splitting into training, test and validation datasets at 80, 18 and 2%, respectively. The different training, test and validation datasets for each cultivar were rejoined after the random split to ensure all cultivars were equally represented in all three datasets. The images were further processed to test whether specific regions of the apple were important in model predictions. The images were processed as above, then they were split in half vertically and horizontally before the quadrants were swapped diagonally (Fig. 2). This caused the outer corners of the apples to be placed in the centre and vice versa.

### Deep learning models

**Fig. 3:**
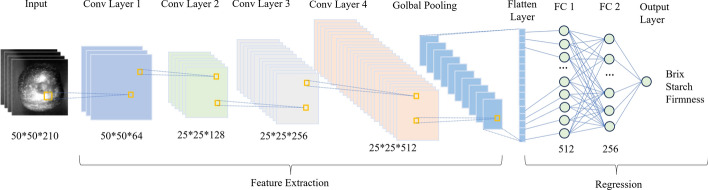
2D-convolutional neural network architecture

**Fig. 4:**
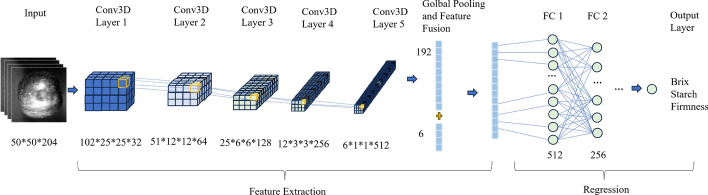
3D-convolutional neural network architecture

**Fig. 5:**
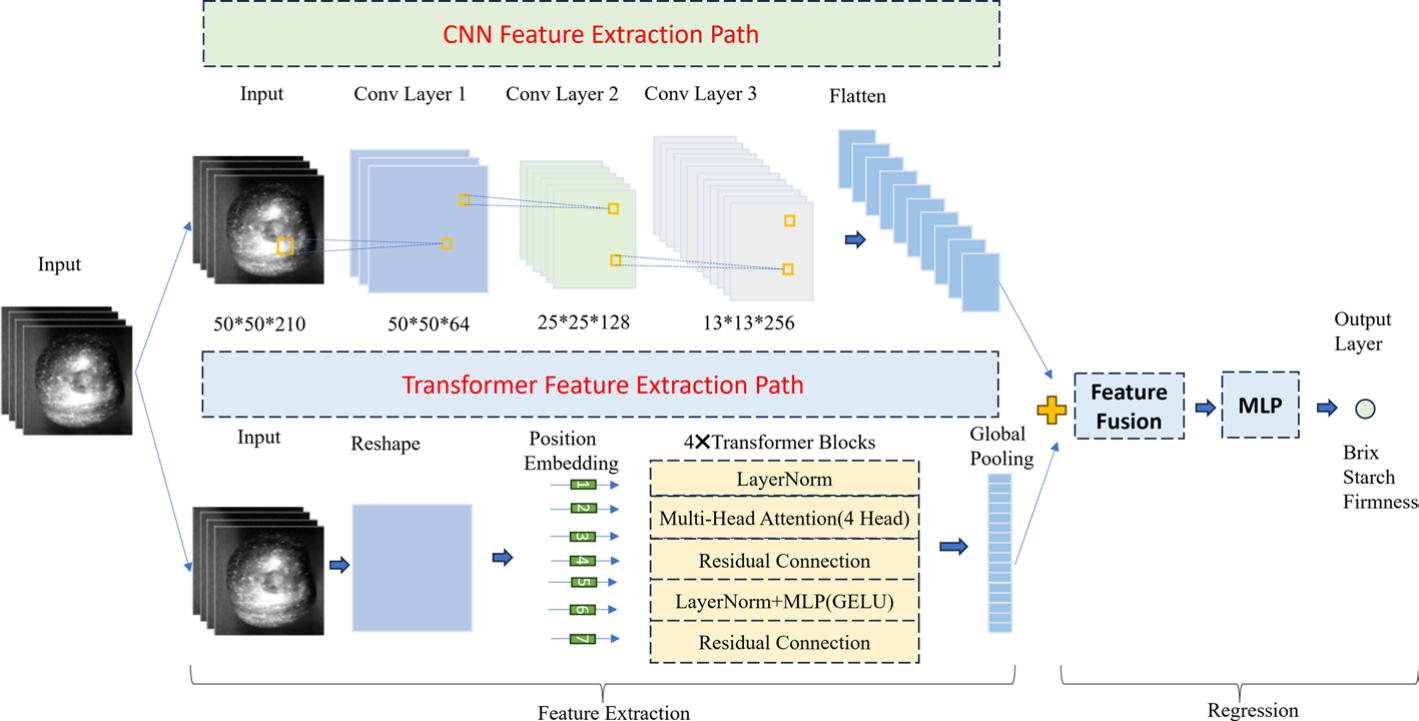
Hybrid model architecture, consisting of 2D-CNN and transformer blocks

**Fig. 6:**
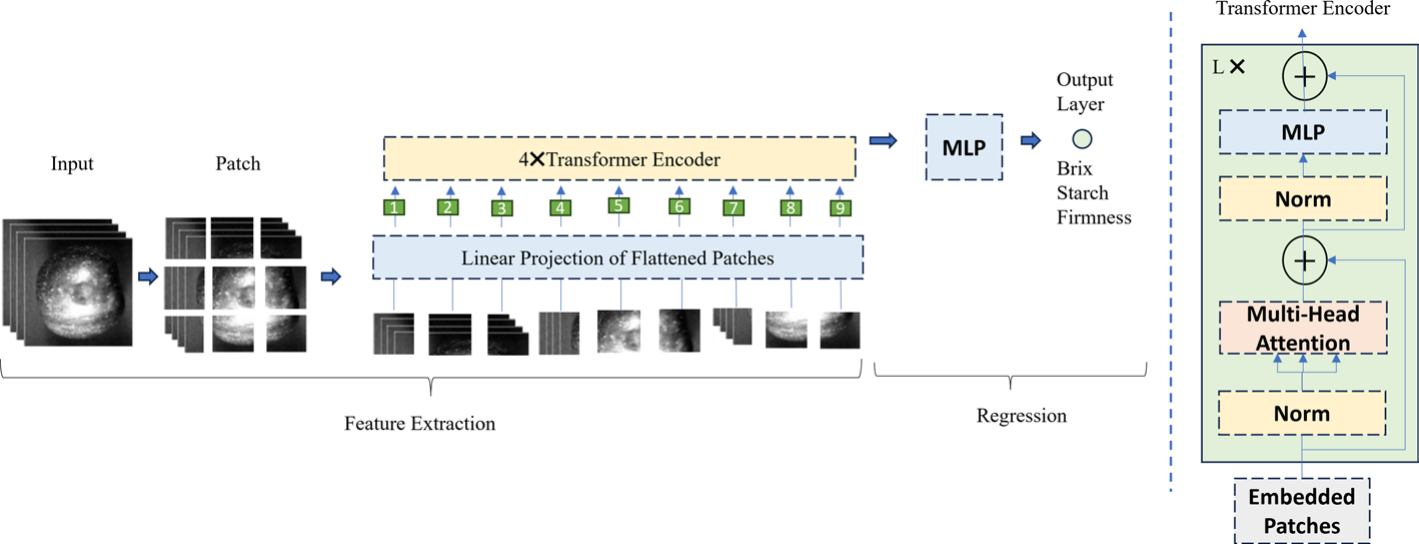
Vision transformer model architecture. Input: input image, Patch: Patch embedding block, MLP: multilayer perceptron, Multi-head attention: multi-head self-attention mechanism, Norm: normalisation layers

Unlike traditional multivariate machine learning models (e.g., PLSR, SVR) that typically utilise 1D averaged spectra, the architectures selected for this study are specifically capable of processing 3D spatial-spectral tensors. This approach was chosen to preserve and analyse the critical spatial heterogeneity of the fruit surface, which conventional 1D models inherently discard. The selection of these four model architectures was intended to systematically compare the performance differences of various deep learning strategies in processing hyperspectral data for the values of Brix, firmness, and starch prediction. Specifically, 2D-Convolutional Neural Networks (CNN) excel at extracting local spatial features (Fig. 3); 3D-CNNs can simultaneously capture joint features in both spatial and spectral domains (Fig. 4); Hybrid CNN-Transformer models attempt to combine the local receptive field capabilities of CNNs with the global dependency modelling capabilities of Transformers (Fig. 5); and Vision Transformer (ViT) models rely entirely on self-attention mechanisms for feature learning (Fig. 6) (Supplementary 1).

All models were implemented on the TensorFlow and Keras frameworks. The process utilised *distribute.MirroredStrategy* from TensorFlow for efficient utilisation of available graphics processing units (GPU). Common training configurations included the use of Adam optimiser and Mean Absolute Error (MAE) as the primary evaluation metric. MAE intuitively reflects the average magnitude of deviation between predicted and true values. During the training process, MAE on the validation set also serves as a key metric for monitoring model performance, making early stopping decisions, and selecting the best model weights (saved via the ModelCheckpoint callback). Concurrently, loss function values (such as MSE or Huber loss) during training will also be recorded and analysed to aid in understanding the model’s convergence behaviour. The CSVLogger recorded a detailed training history.

### Hyperparameter tuning

Bayesian optimisation is a method to optimise model hyperparameters [30]. Bayesian optimisation was used to determine the hyperparameters only for the ViT model, as it demonstrated the strongest baseline performance among the four architectures tested. The hyperparameters and their range are listed as follows: the number of layers (1–5), patch size (2–10), projection dimension (64–256), number of heads (1–5), multilayer perceptron (MLP) head units (small head: [128, 64], medium head: [256–128] or large head: [256–128-64]) and dropout rates (0.1−0.5) were tested. The larger the head, the more complex patterns can be learnt, but it may overfit and require more time to train. These hyperparameter ranges were selected to intentionally span low, medium and high levels to ensure the analysis can capture all possible results with various hyperparameters. The final model was evaluated as follows.

### Model evaluation and feature selection

The three models return outputs for Brix, average firmness and starch percentage, independently. Root Mean Squared Error (RMSE) and the Coefficient of Determination ($R^2$) were used to determine the performance of the model. Evaluation of important wavebands, apple regions of interest and cultivar was done using Shapley values. Shapley value is based on coalition theory to fairly split a prize pool of money, based on their contribution. The greater their contribution, the greater their winnings. We calculate Shapley values for each feature on our final machine learning models to determine the contribution of each waveband to each maturity feature prediction, as well as the significance of each region of the apple and cultivars.

Floating Point Operations (FLOPs) were used to measure the complexity of models [9]. The lower the FLOPs value, the lower the computational cost. We used FLOPs to assess the difference between the Bayesian optimised models and the models trained on default parameters.

### Hardware and software environment

Models were trained and evaluated with Python 3.10.13, using Keras (v2.15.0), Pytorch (v2.1.0), scikit-learn (v1.4.2), Numpy (v1.26.4), Pandas (v2.2.3), OpenCV (v4.8.1) and Matplotlib (v3.8.2). All ML models were trained on a NVIDIA A100 (80 GB), 503 GB RAM system. The code can be found in our Github repository and the dataset in the Essex repository .

### Modelling strategies to assess model performance on maturity features

**Fig. 7:**
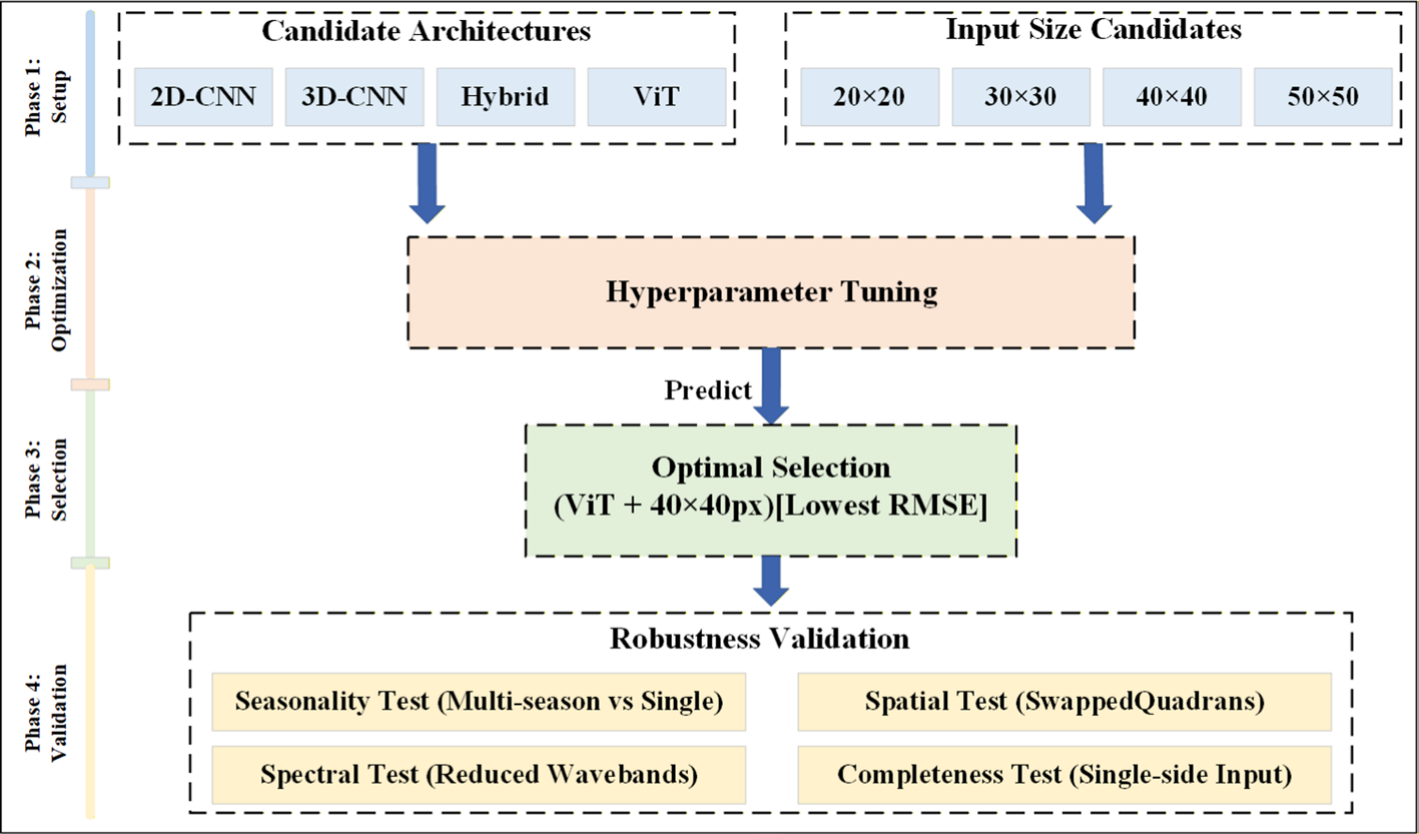
Model training and testing workflow

Several tests were conducted to get the optimised models for Brix, firmness and starch (Fig. 7). We first tested between 2D-CNN, 3D-CNN, Hybrid or ViT models to identify the best performing model type. Secondly, different image sizes (30-by-30 or 50-by-50 pixels) and cropped (20-by-20 or 40-by-40 pixels) images were fed into the best model to determine the best input shape. These models were optimised using Bayesian optimisation functions to tune the hyperparameters of the models for each of the maturity features, independently. On the optimised models, we compared the effects of (1) removing the cultivar encoding, (2) just having one model for all three features and (3) reducing the input to just a single side of the apples (instead of using images from all sides of the fruits). We further tested (4) the effects of segregating the data into their seasons and (5) retaining a percentage of high impact channels and observing the effect on the models. Finally, (6) we tested how important each region of the apple is by splitting apple images into quadrants and swapping quadrants diagonally. All model RMSE and $R^2$ values were compared between the models.

## Results

### Analysis of maturity parameters

**Fig. 8:**
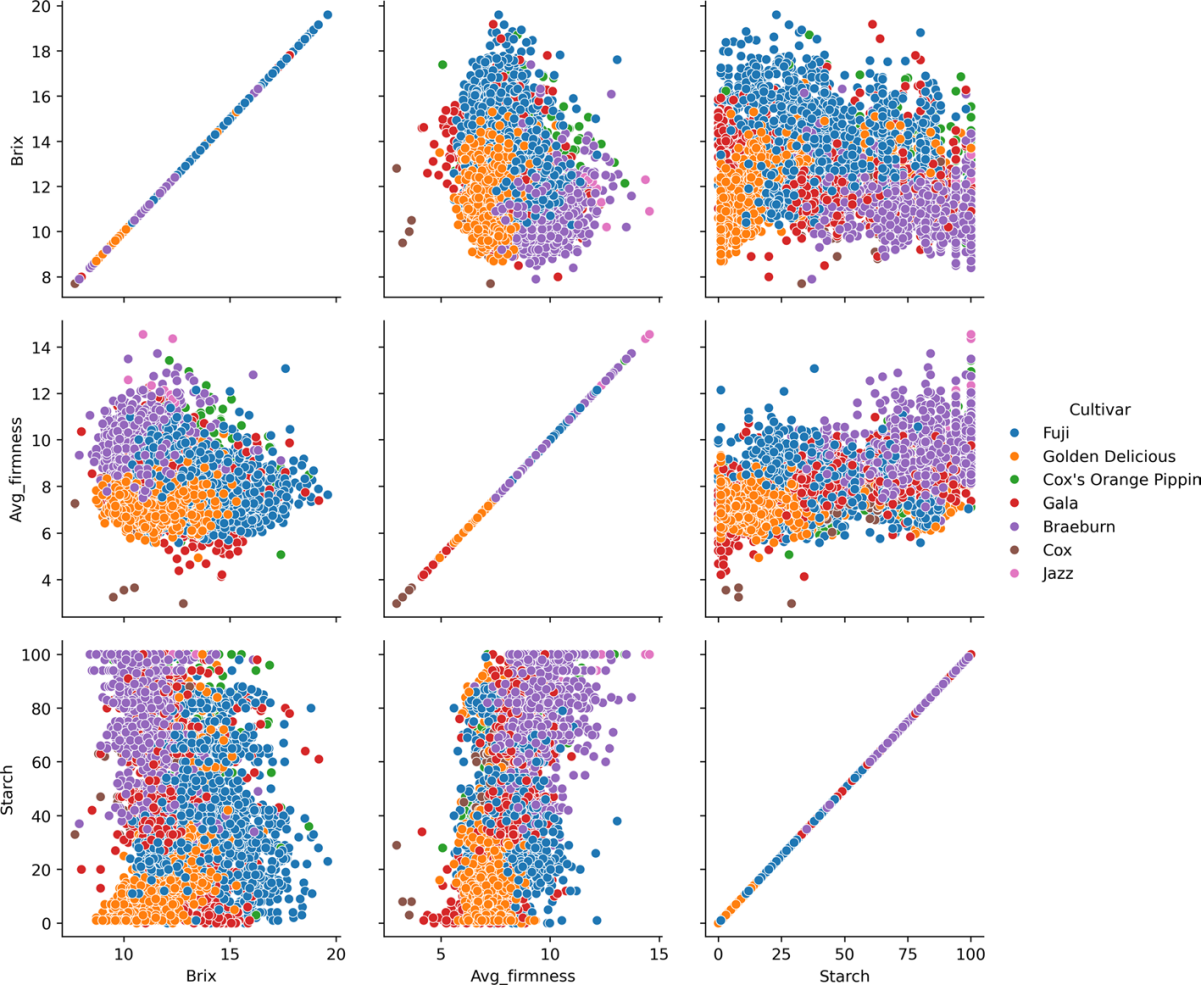
Pairwise plot between Brix, firmness and starch, coloured by each cultivar

Harvests were done over the commercial harvest period for six apple cultivars, and their starch, Brix and firmness measurements were recorded (Table 2). Firmness is measured twice for each fruit. The overall standard deviation of firmness between sides is 0.45 ± 0.38 kgf, with a minimum of 0 and maximum of 2.63 kgf. The fruit with lower firmness was likely to have lower starch levels and vice versa, as seen by the slight positive trend (Fig. 8).

As expected, Brix and firmness are more similar within cultivars than between cultivars. Factorial Analysis of variance for each trait against season and cultivar was conducted to determine the percentage of variance explained by each factor (Table 3). Most of the variation is attributed to cultivar differences, explaining 33%, 22.91% and 25.44% of the variance in firmness, Brix and starch, respectively. The variation due to season had a lesser effect on firmness (9.57%) and starch (1.47%), but it explained a large amount of variance for Brix (21.25%). This indicates that the amount of soluble sugars is largely affected by the climate during the growing season. Water content, which affects both Brix and firmness levels, is likely a significant factor affecting these traits. The variance explained by the interaction between season and cultivar showed a smaller percentage explained by the firmness and Brix models. For starch, the relatively high variance attributed to the interaction between season and cultivar (20.28%) likely reflects differences in the maturity stage at which cultivars were collected across seasons. Overall, although cultivar and season contribute to predicting fruit maturity traits, more than 50% of the variance remains unexplained, suggesting that an image based deep learning approach may be able to capture additional patterns in the data.

**Table 2:** Quality distribution table of apples by cultivar from 2023–2024

Cultivar	Season	Firmness (kgf)	Brix ($^{\circ }$Brix)	Starch (%)
Braeburn	NZ2023	*8.11 ± 0.63*	*12.41 ± 1.0*	*55.00 ± 21.59*
	NZ2024	*9.25 ± 1.01*	*11.97 ± 1.04*	*69.04 ± 22.06*
	UK2024	*9.72 ± 0.89*	*10.58 ± 0.82*	*83.01 ± 15.70*
	All	*9.51 ± 0.98*	*11.07 ± 1.13*	*77.73 ± 19.71*
Cox’s orange pippin	NZ2023	*8.36 ± 1.36*	*13.02 ± 1.49*	*67.59 ± 22.25*
	UK2024	*8.06 ± 1.25*	*10.83 ± 0.95*	*62.18 ± 15.59*
	All	*8.26 ± 1.33*	*12.28 ± 1.69*	*65.76 ± 20.39*
Fuji	NZ2023	*7.45 ± 0.82*	*14.36 ± 1.54*	*32.64 ± 23.95*
	NZ2024	*7.39 ± 0.81*	*14.65 ± 1.41*	*49.07 ± 23.36*
	UK2024	*9.01 ± 0.87*	*12.32 ± 1.12*	*36.83 ± 18.26*
	All	*7.71 ± 1.03*	*14.09 ± 1.66*	*38.67 ± 23.99*
Gala	NZ2023	*7.78 ± 1.10*	*13.15 ± 1.35*	*39.10 ± 28.28*
	NZ2024	*7.81 ± 0.84*	*12.46 ± 1.23*	*44.36 ± 28.41*
	UK2024	*8.77 ± 0.81*	*11.05 ± 0.80*	*69.81 ± 22.06*
	All	*8.27 ± 1.02*	*11.96 ± 1.42*	*55.27 ± 29.18*
Golden delicious	NZ2023	*7.58 ± 0.97*	*11.92 ± 0.72*	*84.27 ± 12.41*
	NZ2024	*7.59 ± 0.61*	*13.16 ± 0.94*	*53.06 ± 18.16*
	UK2024	*7.19 ± 0.68*	*11.62 ± 1.27*	*15.15 ± 14.62*
	All	*7.30 ± 0.75*	*11.88 ± 1.27*	*30.66 ± 29.83*
Jazz	UK2024	*11.35 ± 1.50*	*11.23 ± 0.97*	*95.16 ± 3.83*

The columns represent the mean and standard deviation of the mean for firmness, Brix and starch. Firmness is the average firmness of the two sides of the fruit. The rows represent the six apple cultivars in this study. Each cultivar is calculated for each season separately and with all seasons together

**Table 3:** Summarised ANOVA results for firmness, Brix and starch

Trait	Term	sumsq	Percentage explained
Firmness	Season	2844.18	9.57
	Cultivar	9809.51	33.00
	Season:Cultivar	1749.63	5.89
	Residuals	15321.07	51.54
Trait	Term	sumsq	Percentage explained
Brix	Season	11519.74	21.25
	Cultivar	12423.32	22.91
	Season:Cultivar	1535.15	2.83
	Residuals	28745.06	53.01
Trait	Term	sumsq	Percentage explained
Starch	Season	275,544	1.47
	Cultivar	4,766,826	25.44
	Season:Cultivar	3,799,794	20.28
	Residuals	9,893,828	52.81

The analysis was conducted excluding Cox’s Orange Pippin (collected only in a single season) and Jazz (small sample size) data to preserve the reliability of the results

### Analysis of spectral data

**Fig. 9:**
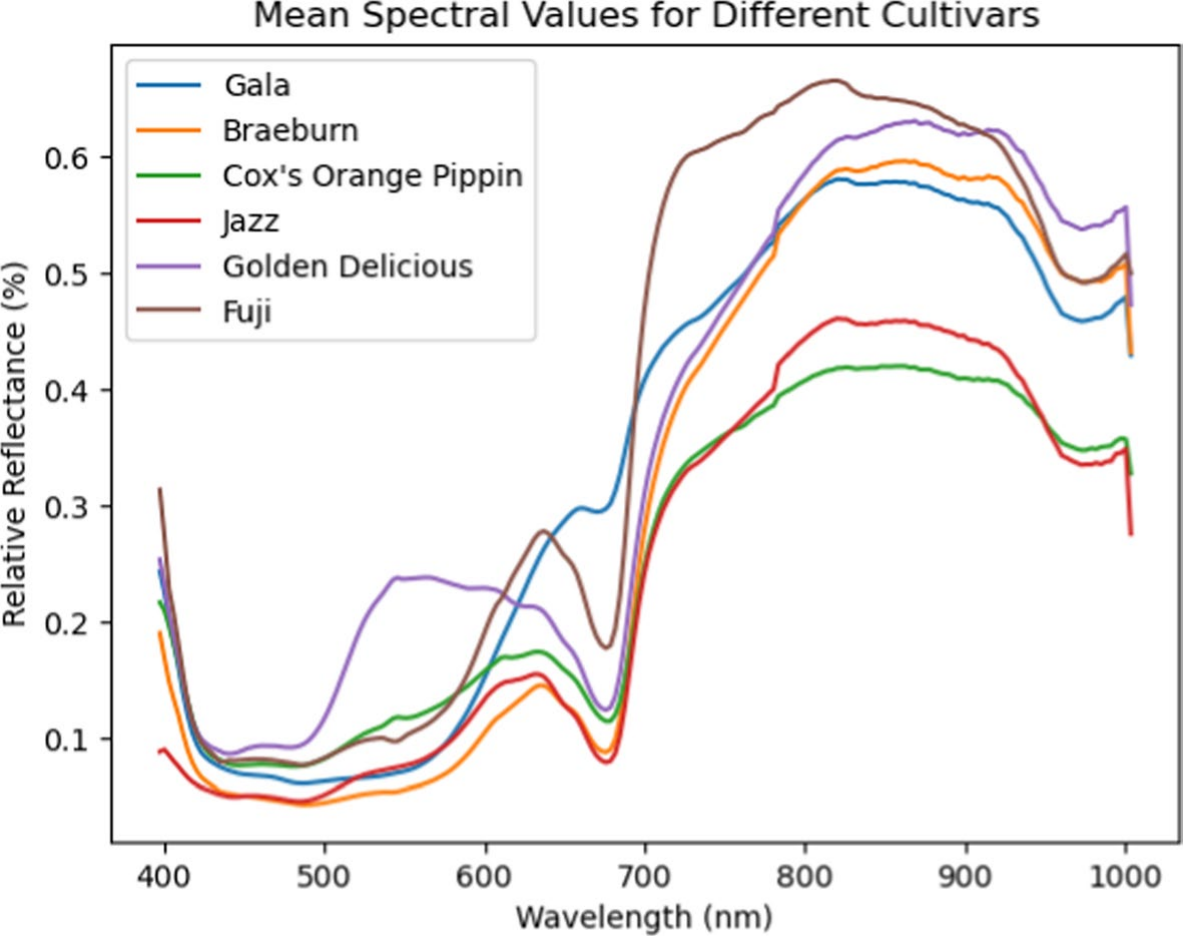
Mean reflectance of six apple cultivars between 400–1000 nm

Figure 9 represents the statistical mean reflectance curves for all six cultivars. The main trends in the spectra are similar between cultivars, namely peaks at 500–700 nm which are coloured pigments such as carotenoids, chlorophyll and anthocyanins; and a large peak at 700 nm which continues onwards to 1000 nm, the limit of the camera. The relative reflectance was distinct for each cultivar, indicating that cultivar types can be inferred through imaging, specifically between 800–900 nm. Blue light, between 400–480 nm, is where carotenoids and chlorophyll absorb light. This is consistent with our results as Golden Delicious has the highest reflectance in this region and Braeburn and Jazz had the least reflectance in this range. Golden Delicious shows a high reflectance peak around 530–640 nm, likely due to the low levels of carotenoid and anthocyanin present in the skin [11]. Gala likely has the lowest concentration of chlorophyll due to the higher reflectance at 650–670 nm [11]. Braeburn and Jazz contain the highest levels of anthocyanins in the peel, as evident by the low reflectance between 550 and 600 nm. Due to significant differences in wavelengths between cultivars, we encoded the cultivar information in the training process.

### Model training analysis

This study tested which type of model was the best in predicting fruit maturity (Table 5; runs 1–4). 2D-CNN, 3D-CNN, Hybrid-Transformer and ViT models were tested on 50-by-50 px images. The best performing model was the ViT model, consistently resulting in the lowest RMSE values for Brix, firmness and starch and the highest $R^2$ values. In comparison, the 2D-CNN, Hybrid and 3D-CNN models were not as accurate as the ViT model. Interestingly, the 3D-CNN model performed better in predicting firmness and starch compared to the Hybrid model, but the Hybrid model did better in predicting Brix. The 2D-model worked well for Brix, ranking second after the ViT results. Firmness and starch were slightly worse than results from the 3D-CNN model but better than the Hybrid model. The second test determined the best size of the data input (Table 5; runs 4–7). The images were aggregated into 30-by-30 pixel or 50-by-50 pixel size. The aggregating process would average across more pixels on larger apples such as Braeburn, compared to smaller apples such as Cox’s Orange Pippin due to the differences in the original apple sizes (Table 4). We further tested 20-by-20 pixel and 40-by-40 pixel images, which were created by removing 5 pixels from each edge of the original 30-by-30 or 50-by-50 pixel images, respectively. This causes the models to be trained only on the central part of the apple face, as the edges may have reduced signal quality [28]. The images sized 40-by-40 pixels, showed the best results for Brix with the edges removed, despite a slight reduction in model performance in firmness and starch. Between 20-by-20 and 40-by-40 pixels sized apples, Brix and firmness had a small improvement in accuracy, but firmness accuracy dropped by a small amount. Therefore, we selected 40-by-40 pixels images to train our subsequent models.

**Table 4:** Mean dimensions of apple cultivars in pixels

Cultivar	Mean height (px)	Mean width (px)
Braeburn	130	136
Cox’s orange pippin	118	122
Fuji	123	131
Gala	124	132
Golden delicious	125	132
Jazz	120	119

**Table 5:** Model summary results for Brix, Firmness, and Starch

					Brix		Firmness		Starch	
Run	Name	Image size	Model	Result	RMSE	$R^2$	RMSE	$R^2$	RMSE	$R^2$
1	Model type	50px	2D-CNN	Runs 1–4: Vision transformer model outperformed the other model types	1.14	0.61	0.86	0.53	19.89	0.57
2	Model type	50px	3D-CNN		1.97	−0.16	1.15	0.15	20.46	0.54
3	Model type	50px	Hybrid		1.33	0.47	1.78	−1.03	34.70	−0.31
4	Model type	50px	ViT	Runs 4–7: Results are slightly better with edges removed (either 40 or 20 px images). 40px chosen for subsequent processes	0.94	0.74	0.74	0.65	17.33	0.67
5	Image size	40px	ViT		0.92	0.75	0.76	0.63	17.37	0.67
6	Image size	30px	ViT		0.98	0.71	0.75	0.64	17.79	0.66
7	Image size	20px	ViT		0.95	0.73	0.76	0.63	17.11	0.68
8	Optimised hyperparameters	40px	ViT	Run 5 versus 8: Optimised hyperparameters with Bayesian optimisation function. Results improved or unchanged for all features	0.91	0.75	0.76	0.63	16.79	0.69
9	Without cultivar	40px	ViT	Run 9 versus 8: Embedded cultivar data improved model results for all features	0.97	0.72	0.79	0.60	17.53	0.67
10	All 3 features	40px	ViT	Run 10 versus 8: A model for each feature is more accurate than one model for all three features	1.08	0.65	0.92	0.46	17.13	0.68
11	Top 20% wavelengths	40px	ViT	Runs 11–13 versus 8: Retained the specified amount of channels and retrained the models. Retaining 50% of the data was as good as modelling with all channels	0.94	0.73	0.76	0.63	18.49	0.63
12	Top 50% wavelengths	40px	ViT		0.89	0.76	0.76	0.63	17.31	0.67
13	Top 80% wavelengths	40px	ViT		0.89	0.76	0.76	0.63	17.07	0.68
14	Swapped corners diagonally	40px	ViT	Run 14 versus 8: Swapped images reduced model accuracy for Brix and starch but did not affect firmness	1.00	0.70	0.76	0.63	18.75	0.62
15	Single side	40px	ViT	Run 15 versus 8: Trained using one side of the apples	0.96	0.72	0.83	0.55	19.24	0.60

**Fig. 10:**
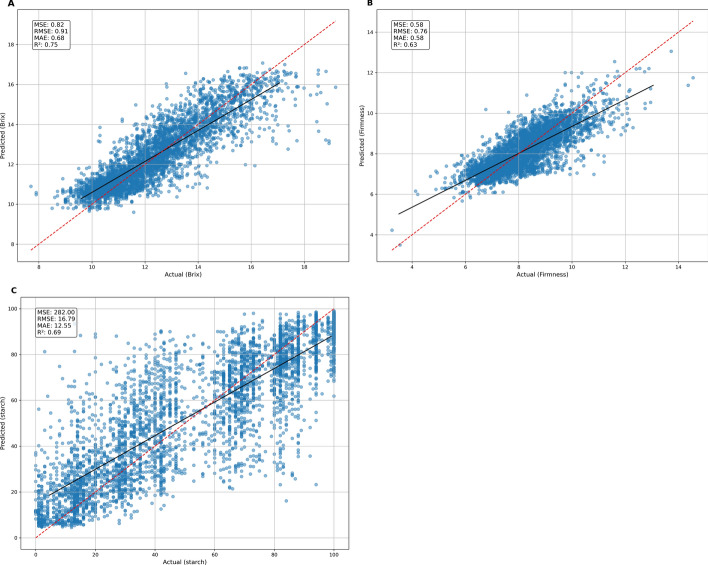
Predicted versus actual values of **A** Brix, **B** firmness and **C** starch on the optimised trained models. The red dashed line represents the x=y line and the black line is the line of best fit

A Bayesian optimisation function was used to find the optimal hyperparameters of the models (Table 6). This changed the number of convolutional blocks (each containing Conv2d, normalisation, max pooling and dropout layers) from 4 blocks to 5 for Brix and 3 for firmness, and the number of blocks for starch was unchanged. The patch sizes were increased after the optimisation for all features, and the number of heads decreased from the default model. The projection dimension increased for Brix but decreased for firmness and starch. The dropout rates also increased for firmness and starch but remained the same for Brix. In terms of MLP heads, Brix and firmness had less layers and similar number of neurons. The MLP head layers and neurons were unchanged for starch. The optimised hyperparameters resulted in models with similar performance for Brix and firmness between runs 5 and 8 (Table 5). Starch results showed significant improvement in RMSE (17.37 vs. 16.79) and $R^2$ (0.67 vs. 0.69) between runs 5 and 8, respectively. Although no large improvements in RMSE or $R^2$ was observed, all optimised models reduced the number of FLOPs required to make model predictions (Table 7). The plot of predicted versus actual values of Brix and firmness show good predictions close to the x=y line, evenly spread across the range with equal deviance on both sides of the line (Fig. 10). For starch, the points follow a vague positive trend, but it has high deviation, especially around the centre of the plot (between 20 and 80%).

**Table 6:** Bayesian Optimisation results for Brix, firmness and starch models

Model	Transformer layers	Patch size	Projection Dim.	Head number	MLP head units	Dropout
Default	4	5	128	8	[256, 128, 64]	0.1
Brix	5	8	256	3	[256, 128]	0.1
Firmness	3	10	64	5	[128, 64]	0.223
Starch	4	9	66	4	[256, 128, 64]	0.137

The default represents the parameters used prior to optimisation

**Table 7:** Floating Point Operations (FLOPs) calculated for each model before and after Bayesian optimisation

Model	Brix	Firmness	Starch
Pre-optimised model	$4.59 \times 10^8$	$4.59 \times 10^8$	$4.59 \times 10^8$
Optimised model	$2.39 \times 10^8$	$4.48 \times 10^7$	$3.85 \times 10^7$

Encoding cultivar data into the last layer of the training data yielded improved results for all three features (Table 5; run 9 vs. 8). We also determined if the performance of a single model, predicting three features (Brix, firmness and starch), would be as accurate as three separate models predicting one feature each. We tested this feature on the 2D-CNN model in preliminary testing (results not shown) and with the ViT model. Model accuracy improved by training three separate models for Brix and firmness compared to a single model (Table 5; runs 8 and 10). There was a significant improvement in model performance with Brix and firmness. For Starch, there were no significant differences. Therefore, the subsequent models trained independent features.

### Seasonal comparisons

To see if the variation in model predictions is due to seasonal variation, the models are trained with each season independently and cross-validated the seasons with each other, computing each five times on five randomly generated test sample subsets and averaging the RMSE (Table 8). Applying one season’s model on another season, in most cases, will result in worse RMSE for Brix and firmness. The exception was the firmness model trained on NZ2024 data and tested on NZ2023 (RMSE = 1.17) data, compared to results on data from the same year (RMSE = 1.28). Overall, we observe less predictive accuracy and correlation between predictions and observed results when models are trained using single seasons for Brix and firmness. The results for starch always produced high RMSE and did not show seasonal specificity.

**Table 8:** Cross-validation of seasonal data on other seasons data

	Data			
		NZ2023	NZ2024	UK2024
Model	**Brix**			
	NZ2023	1.90	2.20	1.89
	NZ2024	2.18	1.98	2.50
	UK2024	2.80	2.76	1.47
	**Firmness**			
	NZ2023	1.16	1.30	1.60
	NZ2024	1.17	1.28	1.62
	UK2024	1.98	1.92	1.47
	**Starch**			
	NZ2023	36.63	32.06	32.25
	NZ2024	32.80	26.51	38.55
	UK2024	36.27	36.27	38.73

The values are the average RMSE computed across five randomly sampled subsets of the test dataset

### Single-sided analysis

**Fig. 11:**
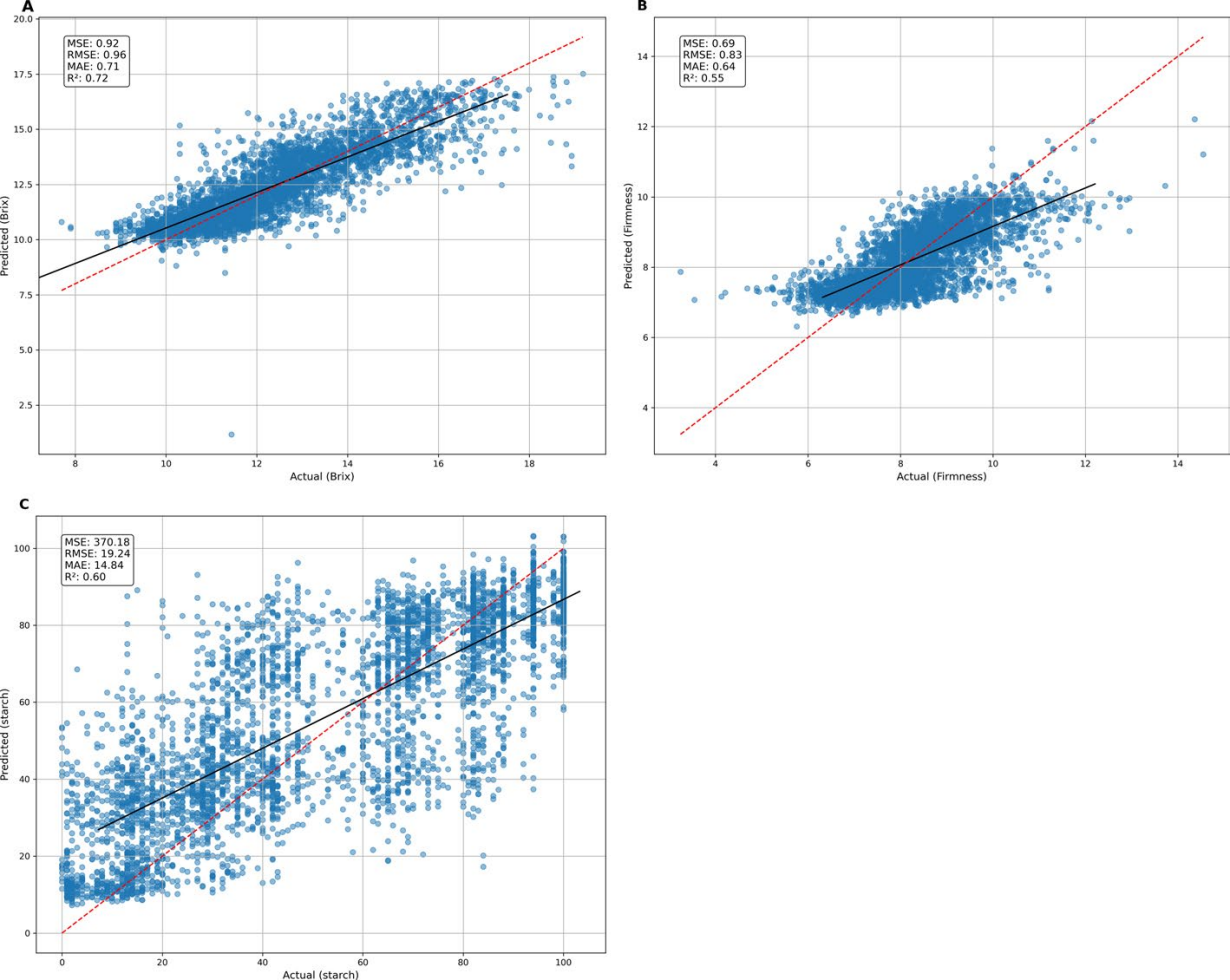
Prediction versus actual plot of **A** Brix, **B** firmness and **C** starch models on a single side of apple data. The red dashed line represents the x=y line and the black line is the line of best fit

Finally, we tested whether using a single side of the apple would yield accurate results. The model was trained on one of the four sides of the imaging data and its corresponding maturity data. The models showed lower accuracy for all features (Fig. 11). The tails of Brix and firmness plots start to flatten out, indicating some bias in the models. The shape of the firmness points is less defined and appears to follow less of a positive trend. Starch remains just as variable as with all four sides of the apple data.

**Fig. 12:**
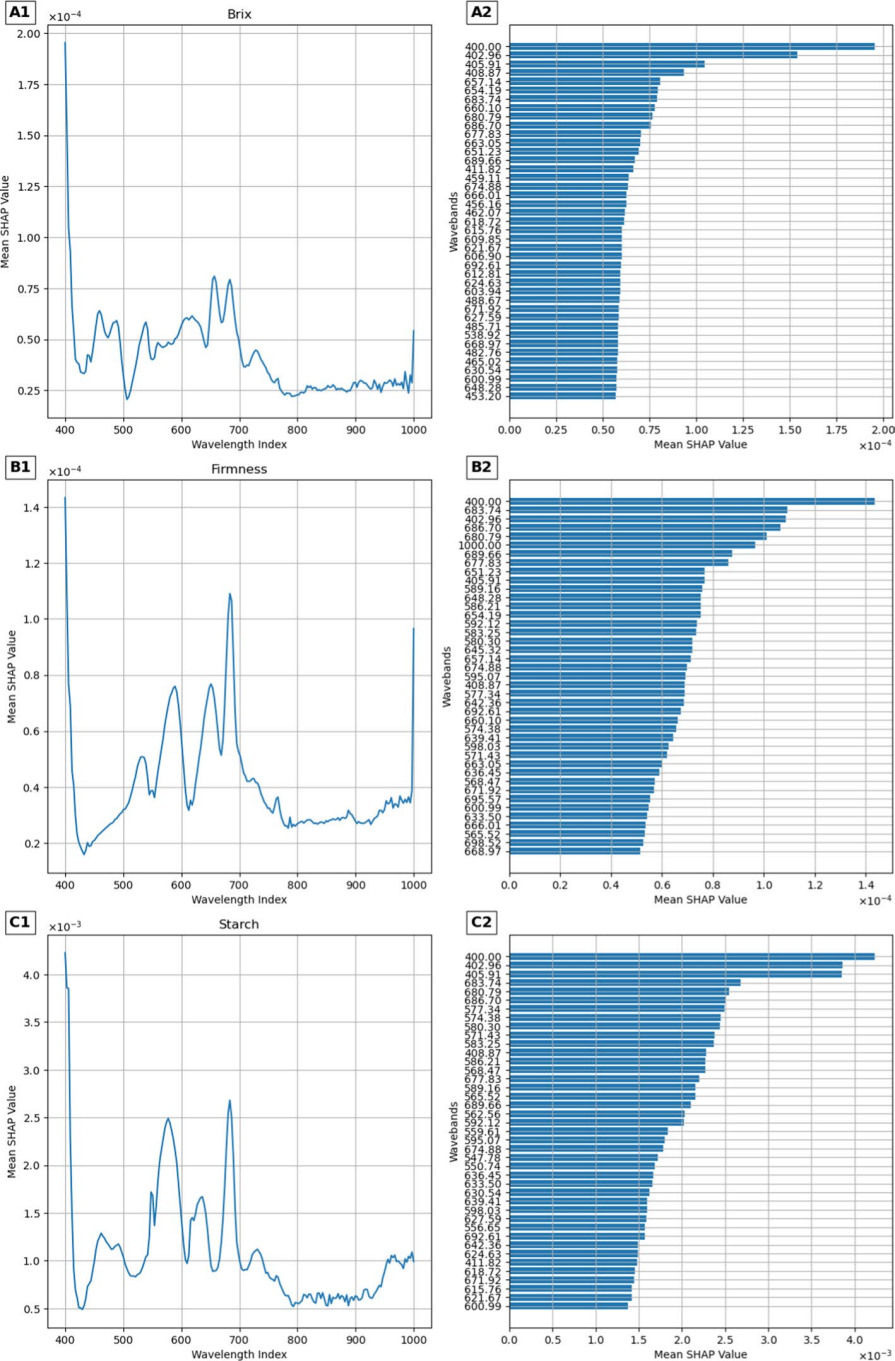
Mean spectral importance. (1) Mean Shapley values across wavebands and (2) the top 20% most significant wavebands for the **A** Brix, **B** firmness and **C** starch models

### Shapley analysis

**Fig. 13:**
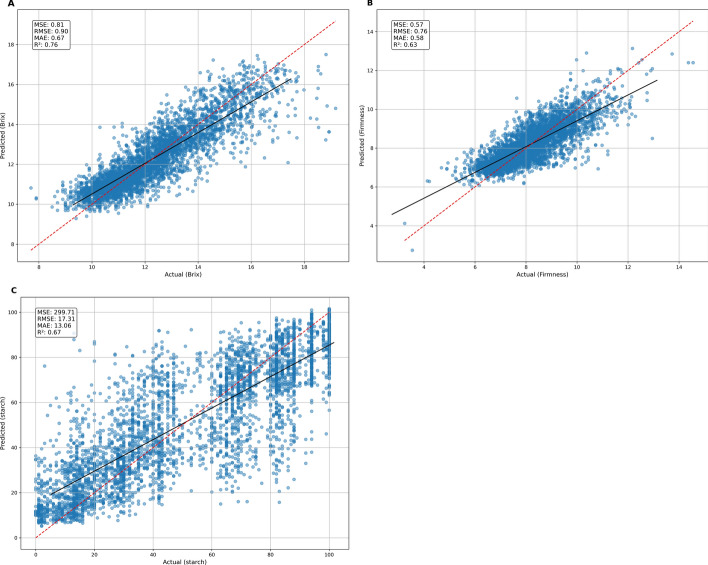
Predicted versus actual plot of **A** Brix, **B** firmness and **C** starch models trained with 50% reduced wavelengths. The red dashed line represents the x=y line and the black line is the line of best fit

Hyperspectral data are highly correlated, making a large portion of the channels redundant. Shapley values indicate that the top 20% of the most important spectral channels were between 400–411, 453–465, 482–488, 538, 600–630, 648–668 and 671–692 nm (peaking at 400, 459, 488, 538, 618, 657 and 683 nm) for Brix (Fig. 12A); 400–408, 633–698, 565–600 and 1000 nm (peaking at 400, 589, 651, 683, and 1000 nm) for firmness (Fig. 12B); and 400–411, 547–598, 600–642, 671–692 nm (peaking at 400, 547, 577, 636 and 683 nm) for starch (Fig. 12C). The spectra between 800–950 nm had small Shap values, indicating a low level of importance of wavelengths in this region. Using Shap values as the level of importance, we kept 20%, 50% and 80% of the most important channels for each feature and retrained the models with the reduced channels. The results show that reducing the wavelength channels to the top 20% for firmness does not affect model accuracy (RMSE = 0.76 and $R^2$ = 0.63). For Brix, reducing the channel wavelengths to the top 50% slightly improves model accuracy (RMSE = 0.89 and $R^2$ = 0.75). For starch, even keeping the top 80% of channels causes a reduction in model accuracy (RMSE = 17.07 and $R^2$ = 0.68). Removing 50% of the wavelengths does not induce any noticeable bias in model predictions (Fig. 13).

**Fig. 14:**
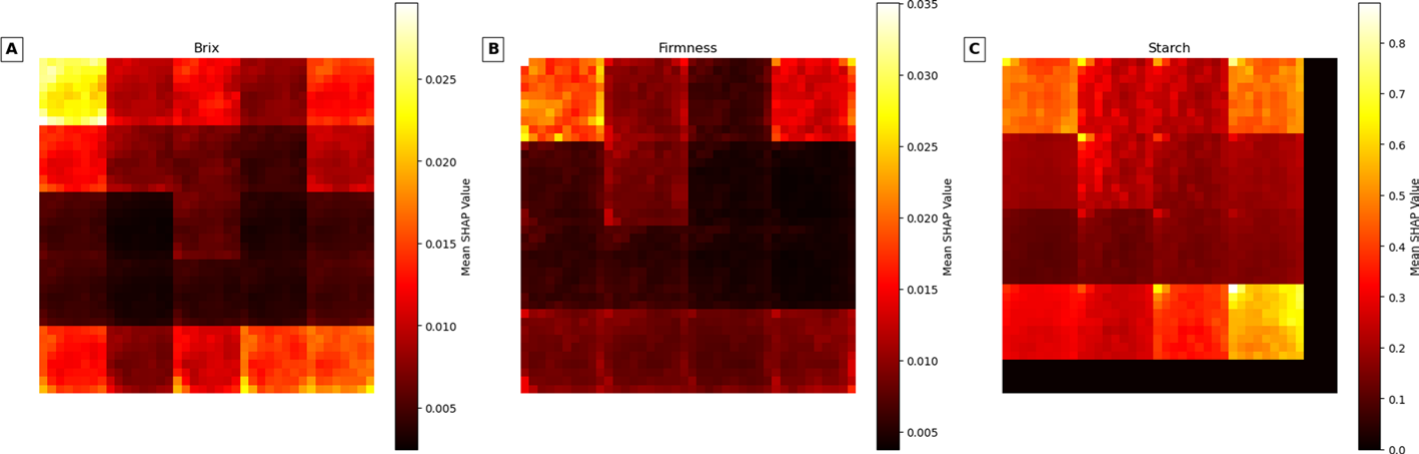
Mean spectral importance (Shapley values) per pixel for all apples for **A** Brix, **B** firmness and **C** starch models. The black margin in starch is due to the image size (40-by-40 pixels) being divided into patch sizes of 9-by-9 pixels

Shapley values were used to evaluate the significance of patches and regions of the apples. The Shapley values indicate that the models put more emphasis on the outer regions of the apple images, in particular the top left corners for Brix and firmness, the bottom right corners for Brix and starch and the top right corner for firmness and starch. Less significance is seen in the middle section of the apple images (Fig. 14).

**Fig. 15:**
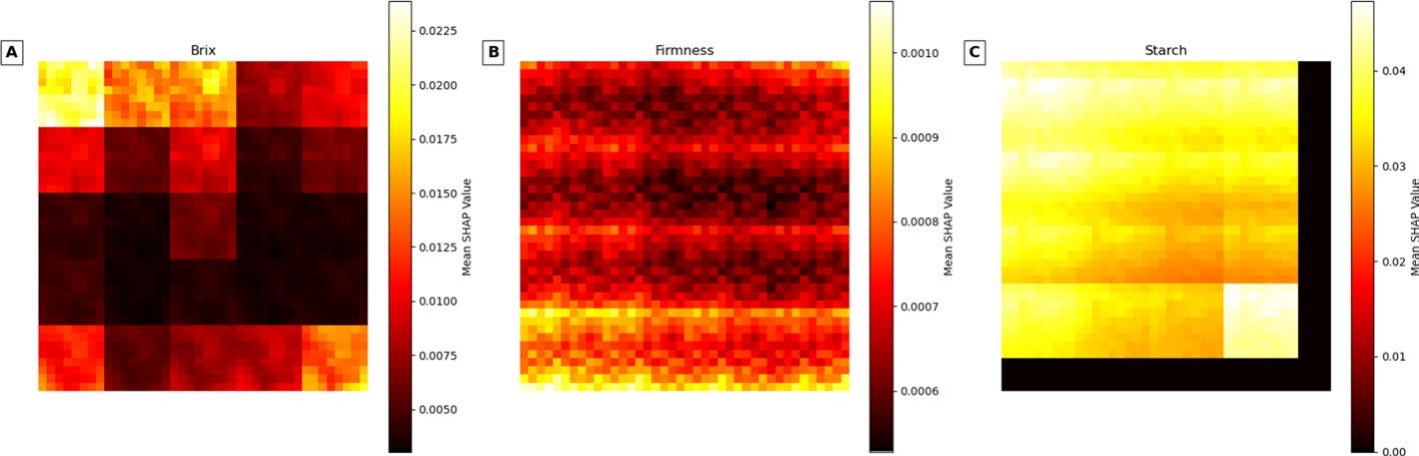
Mean spectral importance (Shapley values) per pixel for all apples for **A** Brix, **B** firmness and **C** starch models trained on swapped images. The black margin in starch is due to the image size (40-by-40 pixels) being divided into patch sizes of 9-by-9 pixels

To test whether this region is significant or whether the models are more attentive to these regions of the data, we trained the model on images with quadrants swapped (Fig. 2). The Shapley values showed very similar patterning in Brix, indicating that the model tends to look at the top left (first patch into the model) and the bottom right (last patch into the model) (Fig. 15). The regions are insignificant for Brix. However, the model prediction accuracy dropped for Brix (RMSE = 1.00, $R^2$ = 0.7) compared to the original images (RMSE = 0.91, $R^2$ = 0.75). The pattern of the Shapley values is altered for firmness, the most significant patches are in the bottom row. However, the degree of significance for firmness is very low (maximum Shapley value is  0.001) compared to Brix (0.023) and starch (0.04). For starch, all patches are now significant, with the middle section having slightly less importance than the outer patches. In particular, the top left and bottom right, as before, were important patches, indicating that the models put more attention on the first and last patches. Starch model accuracy dropped slightly between the original images and swapped images (Table 5; run 8 vs. 17).

## Discussion

In our study, we evaluated the state-of-the-art effective model and tested the effects of wavelengths, regions of interest, cultivar encoding, and seasonality on models predicting Brix, firmness and starch. We found that imaging is an effective method for predicting Brix and firmness, but it does not work well for starch. The best model performance is achieved by training Vision Transformer models for each maturity feature, on datasets from different seasons and geographic locations, training the models on more than one side of the fruit and encoding cultivar information in the training process. Reductions in computational processes can be achieved by reducing the wavelengths to the top 50% of most informative wavelengths.

### Cultivar and seasonal information

The work in the present study incorporates data from multiple seasons, cultivars and geographical locations, in contrast to most previous studies whose dataset limit their generalisability. We assess the effect this additional information has on our model performance. The broader scope introduces more variability into the models, which may partially explain the comparatively poorer performance metrics compared to more focused studies. However, diversity is essential for building robust and generalisable models.

Analysis of variance explained by cultivar and season explained less than 50% of the total variance. Suggesting that cultivar information is an important variable, with a lesser effect by seasonal information. Moreover, the average spectral wavelengths varied distinctly between cultivars thus, cultivars were encoded in the model training process. Without cultivar encoding, we saw a slight drop in model accuracy for all features. During the preliminary assessment, we found that models trained on individual cultivars yielded inaccurate results and were highly bias (data not presented). Similar findings were observed when training individual seasonal data (Table 8). Splitting the training data into smaller sizes typically reduced the model accuracy, a common result in deep learning models. Cross-season prediction tests revealed a degree of season-specificity, with models performing best within their own season. Despite this, the full model (model trained on data from all three seasons) produced the best result, demonstrating the importance of training on diverse datasets to improve consistency and reliability. The authors recommend that data be trained on multi-season and multi-cultivar data for the best model performance.

### Model results

The firmness prediction model performed the best out of the three maturity features tested. We achieved RMSE of 0.76 and $R^2$ of 0.63 (Table 5; run 8). Comparing previous studies from multivariate linear regression (RMSE = 5.76 and $R^2$ = 0.74 [5] and RMSE = 1.26 and $R^2$ = 0.67 [3], least squares support vector machine (RMSE = 4.26 and $R^2$ = 0.88)[5] and partial least squares regression (PLSR) (RMSE = 0.99 and $R^2$ = 0.78) [28], predictions from our deep learning model yielded better RMSE but lower $R^2$ values. Our results are similar to a study using artificial neural network models (RMSE = 0.722 and $R^2$ = 0.896) [19]. Their study recorded firmness at a puncture depth of 1 mm, whereas in our study, we used the gold standard of puncture tests at a depth of 8 mm for our firmness measurements. It is interesting to find similar results because the mean light penetration depth within the visible region—where high importance of wavelengths was found (Fig. 12B)—is 1–2 mm [21]. Our results suggest that HSI with ViT models is an effective method of predicting apple firmness.

Our second-best model was Brix (RMSE = 0.91, $R^2$ = 0.75). Brix is the measurement of soluble sugars in the juice. Compared to other studies using multivariate linear regression—with reported performance RMSE = 0.6 and $R^2$ = 0.76 [5], RMSE = 0.412 and $R^2$ = 0.96 [10] and RMSE = 0.82 and $R^2$ = 0.76 [3]—as well as least squares support vector machine (RMSE = 0.64 and $R^2$ = 0.74; [5]), and PLSR, with RMSE = 0.56 and $R^2$ = 0.79 [6] and RMSE = 0.54 and $R^2$ = 0.90 [28]), our results using ViT models performed worse. We suspect that Brix does not need complex models to make accurate predictions or perhaps it is due to the increased variation as our data is collected from multiple cultivars and seasons.

Starch percentage or the standard starch pattern index is a measurement of starch coverage inside the fruit. Starch degradation starts from the centre of the fruit, near the seeds and therefore, light does not penetrate deep enough for the models to predict accurate starch predictions [8, 21]. This is evident in our study as predictions closer to unripe (100%) or overripe (0%) were more accurate than predictions in between (Fig. 10). Thus, starch cannot be accurately predicted by spectral imaging. Starch results will not be discussed extensively due to the poor results.

Whilst our RMSE and $R^2$ presented in this study may not perform as well as previous studies, our models were trained on much larger and varied datasets, specifically from multiple cultivars and countries. The immense biological, environmental, and inter-cultivar variability introduces substantial real-world noise into our data. Therefore, higher RMSE and lower $R^2$ values are expected from our models.

### Important channels

Shapley values enable us to decode black box systems and identify which wavelengths are important in the model prediction process. The Shapley values suggest that wavelengths in the visible spectra at ranges 400–411, 453–465, 482–488, 538, 600–630, 648–668 and 671–692 nm were within the top 20% of the most significant wavelengths, with peaks at 400, 459, 488, 538, 618, 657 and 683 nm were important for predicting Brix values (Fig. 12A). Similarly, wavelengths between 400–408, 633–698, 565–600 and 1000 nm with peaks at 400, 589, 651, 683, and 1000 nm were within the top 20% of most important wavelengths for firmness (Fig. 12B). The most important wavelengths identified in our study are similar to those identified by Merzlyak et al. [11] in their Plant Senescence Reflectance Index (PSRI; (R678 – R500)/RNIR or (R678 – R500)/R800), which are correlated to fruit ripening onset, those used by Pourdarbani et al. [20] (535–560, 835–855 and 950–975 nm) and [32] (403, 430, 551, 617, and 846 nm) to predict fruit maturity. The detected metabolites are likely chlorophyll, carotenoids and anthocyanins in the visible spectrum and the oxygen-hydrogen bonds abundant in soluble sugars and water in the NIR region [20]. Other studies have also identified several effective spectral ranges for predicting SSC and firmness within the boundaries of important wavelengths identified in our study. For instance, Wang et al. [28] highlighted normal spectral indices around 746/749 nm, while [33] found the range between 704–805 nm to be effective in sugar content predictions. Similarly, Çetin et al. [3] reported that wavelengths at 505, 511, 704 and 689 nm were useful for SSC prediction. [3] attributed ratio combinations between 680, 880, 905 and 940 nm to firmness predictions. Although the wavelengths are not exact, most can also attribute their spectral results to chlorophyll, carotenoids, anthocyanins and O–H bonds for soluble sugars. Despite significant spectra not being exact, they attained similar or better RMSE and $R^2$ compared to our results. These studies select highly important wavelengths to predict their features. Whereas, in our study, we reduced the number of wavelengths by 50% (retaining 102 channels), proving that most wavelengths between 400–1000 nm are redundant for model predictions. Our process does not guarantee the removal of multicollinear wavelengths, potentially retaining wavelengths with similar information.

### Regions of the fruit

The Shapley values in the original images (Fig. 14) suggests that edges, and more specifically, the top left and bottom right patches, are the most important regions when making model predictions. This would be consistent with the findings of Tian et al. [26], as the curvature of the apples affects the signal-to-noise quality of the reflectance. We tested the theory by swapping quadrants of the original images (Figs. 2 and 15) and found that the Shapley quadrants do not change the significant regions for Brix. This suggests that the regions of the fruit are unimportant for Brix. One reason could be that the soluble sugars of the fruit are homogenous throughout the fruit and therefore, sampling from any region will not make a difference to predictions. Another reason why the top left and bottom right patches may be more significant could be because the model is emphasising on the first and last patch in the model. The reduced accuracy with the swapped quadrants suggests that the quality of the image in the centre part of the apple is not as good as the edges, consistent with the findings from Tian et al. [26].

The original images (Fig. 14) showed the same trend for firmness; where the centre patches are less important and more importance is placed on the edges. We anticipated some importance to be placed in the centre as the puncture site when collecting firmness is near the equator of the apple. When the quadrants were swapped (Fig. 15), the pattern of the Shapley values changed significantly. Each firmness patch was deemed important by Shapley values. This could be due to the uneven firmness across the fruit’s surface, or it might be an artefact of the data collection process, specifically, the side of the fruit used for firmness measurements may not correspond to the side shown in the images. This shows that fruit regions may have some significance for predicting firmness. In the future, if imaging were used for predicting firmness, the side corresponding to the image should be used for firmness measurements.

The Shapley analysis indicates that the trained starch percentage model relies on bands in the visible spectral range (Fig. 12) and that they are significantly affected after swapping the image quadrants (Figs. 14 and 15). However, light does not penetrate deep enough to attain a proper starch reading, resulting in either presence or absence readings when the fruit are unripe or ripe, respectively. This suggests that the model trained to predict starch percentage relies on changes on the surface of the apples that are associated with the ripening process and not on starch degradation specifically. The feature changes on the surface could be associated with physiological changes such as skin colour change from green to red/yellow, thickening of the wax layer, or other physiological traits. Regardless, these surface feature changes are more associated with changes in brix and firmness, as these features are visible on the surface. Therefore, models trained on surface features led to poor predictions for starch, but good predictions for brix and firmness.

### Single-sided analysis

In our study, we captured images from all four sides of each apple. We treated each side as an independent apple. Previous studies averaged each side together to create a mean spectral image of the apple [20]. In our study, we treated each side independently because if imaging is applied to an orchard, the imaging system will only capture one side of the fruit. This is our rationale in testing prediction models trained on a single side of the fruit. These models resulted in a reduction in model accuracy and increased bias on the test dataset. The increased error might be due to a decreased amount of training data. This is common in deep learning models. Another reason could be due to a difference in the side of the image and the corresponding side used to measure firmness. We were unable to match up the sides as it was not recorded during the collection process. The variation of firmness between two sides of the fruit deviates by 0.45 ± 0.38 kgf, which may explain the decrease in model accuracy. To improve model results, the same face should be used to measure Brix and firmness as the side imaged.

### Interpretation of ViT performance superiority

The experimental results in Section 3 consistently demonstrated that the Vision Transformer (ViT) architecture achieved the highest predictive accuracy for apple maturity traits. The fundamental reason for this superiority, when compared to the CNN-based models, lies in the differing receptive fields and feature aggregation mechanisms intrinsic to each architecture. Standard CNNs, including the 2D and 3D variants evaluated in this study, rely on discrete convolutional kernels that focus on local spatial neighbourhoods. This "inductive bias" of translation invariance is useful for general image processing, but it can be restrictive when analysing maturity-related biochemical traits (like Brix) that may have complex, global distributions across the entire fruit surface. Furthermore, traditional CNNs treat spectral bands as independent feature channels or localised dimensions, limiting their ability to capture long-range spectral dependencies. Conversely, the ViT model employs a Multi-head Self-Attention (MSA) mechanism as its core block. The critical functional advantage of this block, as visualised in the Input Embedding Module and Core Transformer Encoder Block in Fig. 6, is its ability to establish global context. Every patch in the image sequence is allowed to attend to every other patch across the entire surface and all spectral bands, dynamically weighting their importance based on the specific prediction task. This allows the ViT to capture intricate non-linear interactions among distant spatial locations and non-adjacent wavelengths that a local CNN would inherently miss. Given the sufficient size of our dataset (5,756 samples), the ViT architecture was able to leverage this high model capacity to learn these sophisticated global features, leading to significantly lower RMSE values.

### Pre-processing method

Fruit maturity is largely a factor of fruit size, shape and colour when discerned with the human eye. However, by locally averaging and aggregating pixels together, it removes size and shape information and averages out skin colour. The models chosen in our study required the same size input. Newer models that allow variable input shapes are available, and it may be worth retraining the models in the current study with different-sized images. In a practical setting within an orchard, retaining a fixed distance between the camera and the apples would be difficult; thus, the current models would achieve better results. There are advantages and disadvantages to either approach. Choosing between any approach would depend on the subject of interest. Patterns on the skin surface may blur but general colouration will be accounted for in the model.

### Applications

As imaging is a non-destructive process, it enables repeated measurements over time. This allows growers to monitor apple maturity progression over the season. Growers can therefore schedule harvests based on the progression of each fruit. Such temporal tracking could significantly enhance crop management strategies, reduce waste and improve fruit quality at market. However, more research is required to adapt the present model to in-field applications. Environmental factors such as variations in light intensities and angles of the light can influence spectral images. Understanding and compensating for these effects are essential for producing a robust model for in-field maturity tracking.

### Limitations

As apples have a curved surface, the light reflection and the distance of the apple edge changes. This results in variable pixel quality, particularly near edges, where shadows or glare may be more pronounced. Such variability can affect the accuracy of model training and introduce bias. In the future, incorporating a curvature-aware preprocessing method may enhance pixel uniformity and improve model accuracy.

## Conclusion

This study focuses on training deep learning models with a large-scale hyperspectral dataset comprising of 5756 apples from multiple countries, seasons and cultivars, providing a robust foundation for predicting apple maturity features. Its scale and diversity mark a significant step forward in the development of reliable, data-driven approaches for non-destructive maturity assessments. By enabling continuous, non-destructive monitoring of fruit maturity, the presented method allows growers to optimise harvest scheduling and automate quality sorting. At an industrial scale, this precise quality control significantly minimises post-harvest food wastage during long-term storage and reduces reliance on labour-intensive manual sampling. Furthermore, ensuring consistent, premium fruit quality enhances a country’s export competitiveness and market value in the global fresh produce industry, thereby providing a tangible boost to the national agricultural economy.

The study determined that (1) ViT models performed that best of the four model architectures selected for this study, resulting in Brix (RMSE = 0.91, $R^2$ = 0.75) and firmness (RMSE = 0.76, $R^2$ = 0.63) predictions within reasonable accuracy. This demonstrates their potential as effective tools for non-destructive apple maturity assessment across diverse cultivars, seasons and countries. Wavelengths important for Brix and firmness predictions were focused around the visible spectrum, with some significant wavelengths around 1000 nm. This indicates that we can reduce the wavelengths captured for the models, resulting in simpler, cheaper cameras that utilise narrower spectra, making imaging will be more accessible to growers. (2) Apple regions were not important when making predictions. Therefore, it is highly likely that when making predictions with obscured fruit, as long as part of the fruit is detected, the model may still predict accurately. Further testing should be conducted. (3) Cultivar and seasons were expected to affect model results as cultivar phenotypes are distinct and can change between seasons depending on the weather from that season. Cultivar encoding appeared to improve model results and seasonal difference were observed but the full model (model trained on all three years of data) yielded the best results.

## Data Availability

The datasets generated during and analysed during the current study are available in the University of Essex repository (https://researchdata.essex.ac.uk/228/) and the code used for data cleaning, model training and analysis are available on GitHub: (https://github.com/EIS-Ressearch-Lab/Apple_maturity_hyperspectral_imaging.git).
